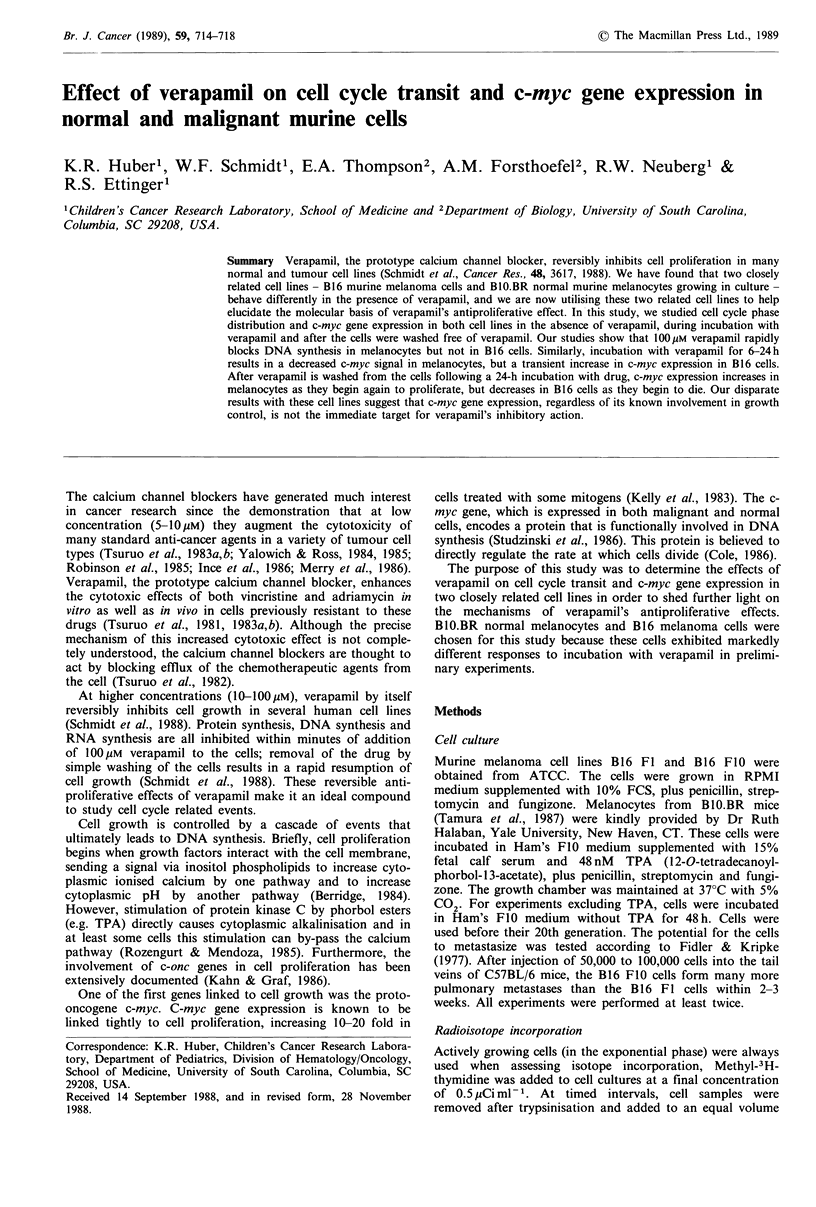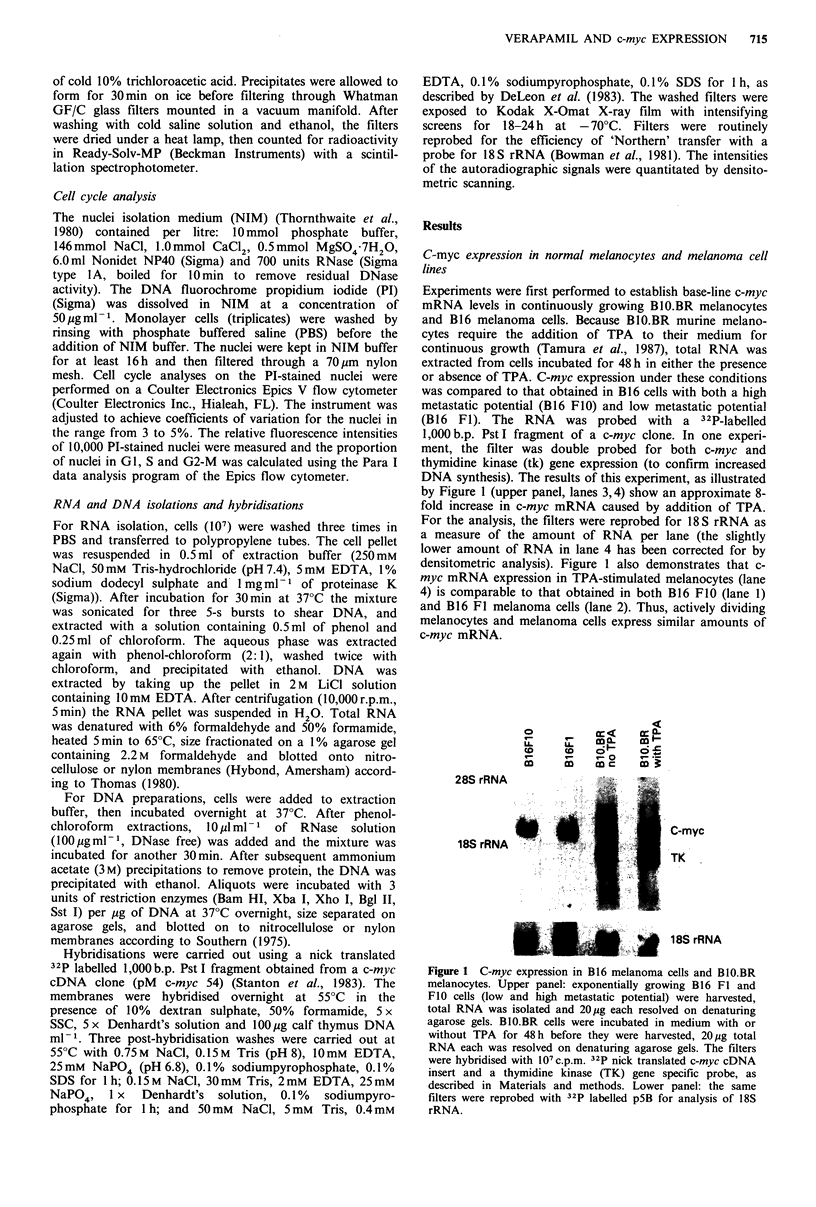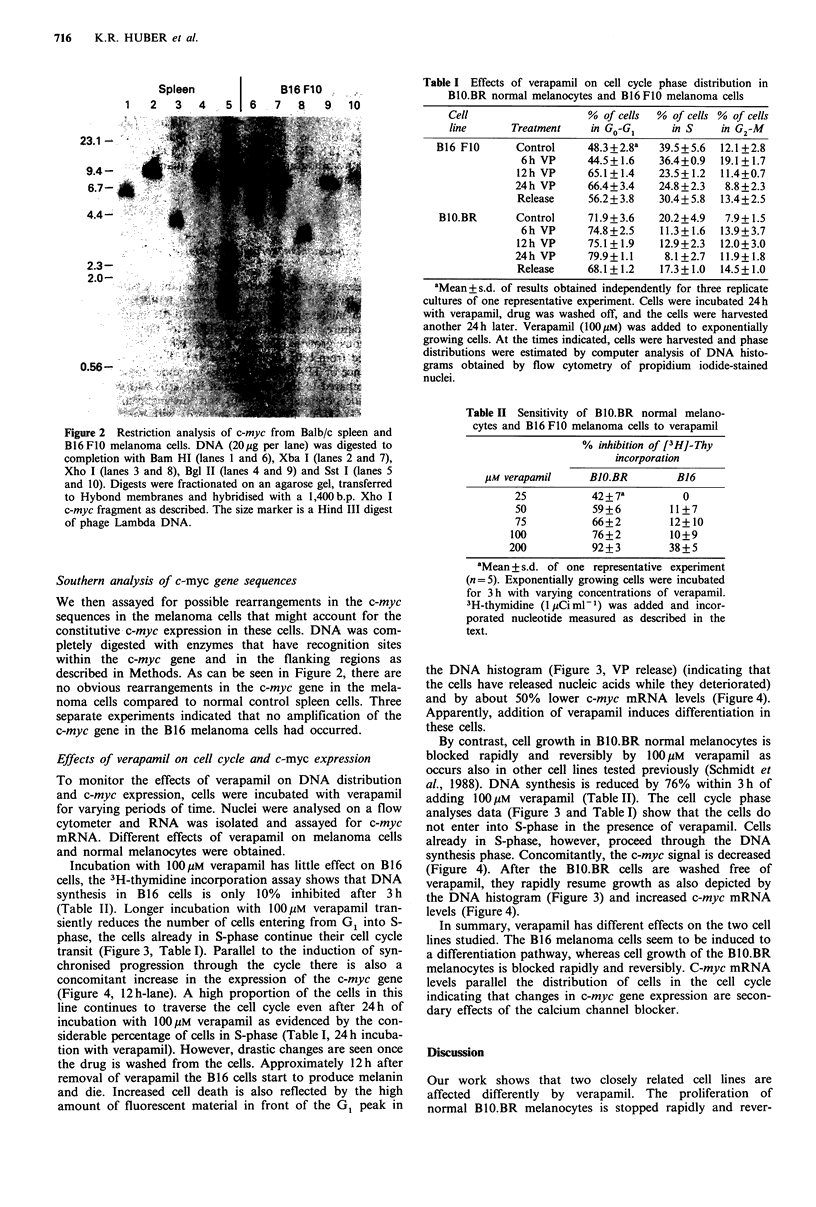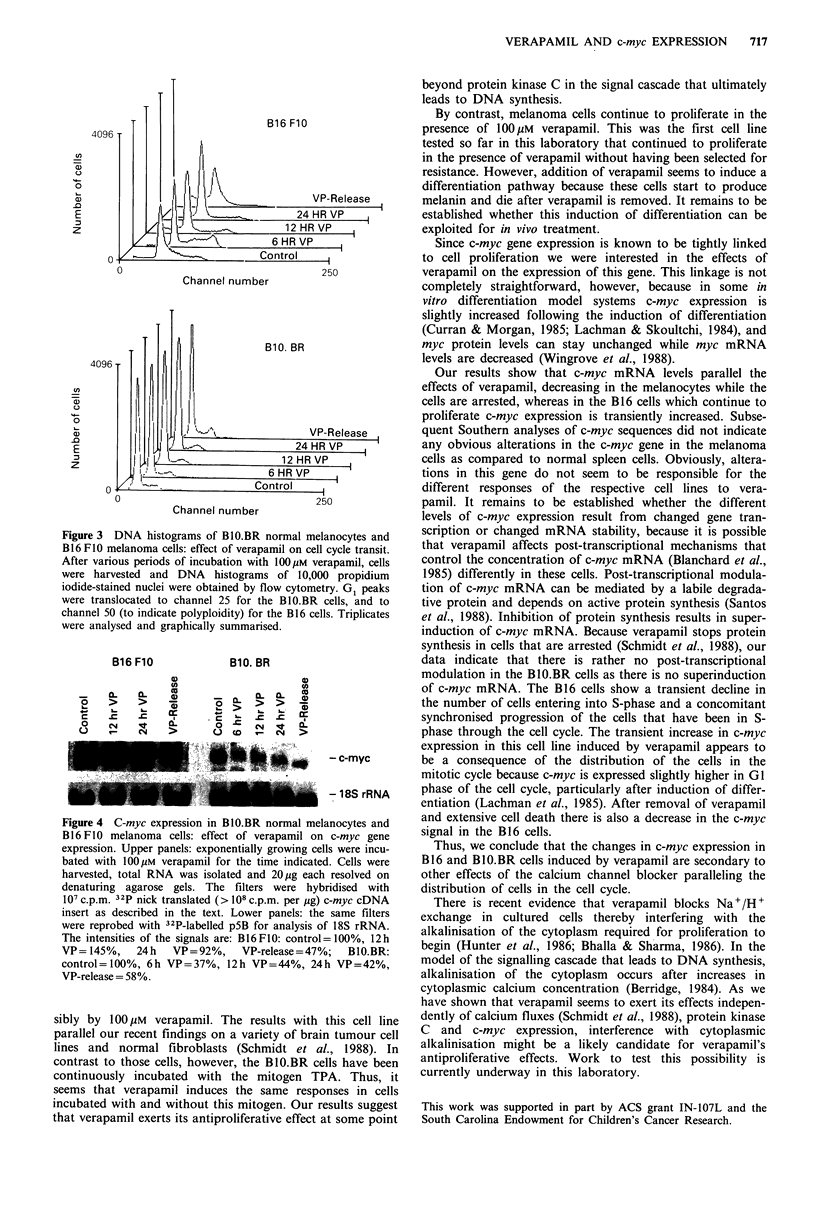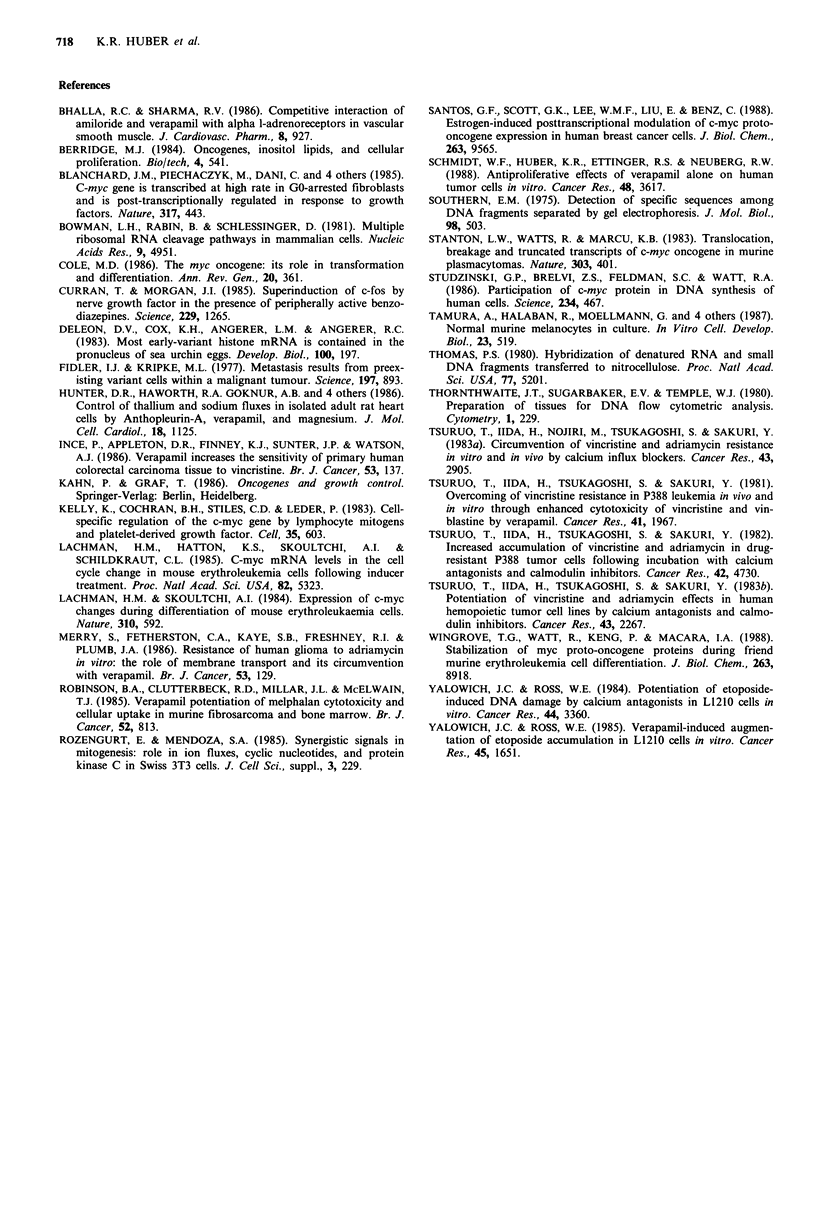# Effect of verapamil on cell cycle transit and c-myc gene expression in normal and malignant murine cells.

**DOI:** 10.1038/bjc.1989.150

**Published:** 1989-05

**Authors:** K. R. Huber, W. F. Schmidt, E. A. Thompson, A. M. Forsthoefel, R. W. Neuberg, R. S. Ettinger

**Affiliations:** Children's Cancer Research Laboratory, School of Medicine, University of South Carolina, Columbia 29208.

## Abstract

**Images:**


Br. J. Cancer (1989), 59, 714–718 © The Macmillan Press Ltd., 1989

### Effect of verapamil on cell cycle transit and c-*myc* gene expression in normal and malignant murine cells

K.R. Huber[1], W.F. Schmidt[1], E.A. Thompson[2], A.M. Forsthoefel[2], R.W. Neuberg[1] & R.S. Ettinger[1]

[1]*Children's Cancer Research Laboratory, School of Medicine and* [2]*Department of Biology, University of South Carolina, Columbia, SC 29208, USA.*

**Summary** Verapamil, the prototype calcium channel blocker, reversibly inhibits cell proliferation in many normal and tumour cell lines (Schmidt *et al.*, *Cancer Res.*, **48**, 3617, 1988). We have found that two closely related cell lines – B16 murine melanoma cells and B10.BR normal murine melanocytes growing in culture – behave differently in the presence of verapamil, and we are now utilising these two related cell lines to help elucidate the molecular basis of verapamil's antiproliferative effect. In this study, we studied cell cycle phase distribution and c-*myc* gene expression in both cell lines in the absence of verapamil, during incubation with verapamil and after the cells were washed free of verapamil. Our studies show that 100 $\mu$M verapamil rapidly blocks DNA synthesis in melanocytes but not in B16 cells. Similarly, incubation with verapamil for 6–24 h results in a decreased c-*myc* signal in melanocytes, but a transient increase in c-*myc* expression in B16 cells. After verapamil is washed from the cells following a 24-h incubation with drug, c-*myc* expression increases in melanocytes as they begin again to proliferate, but decreases in B16 cells as they begin to die. Our disparate results with these cell lines suggest that c-*myc* gene expression, regardless of its known involvement in growth control, is not the immediate target for verapamil's inhibitory action.

The calcium channel blockers have generated much interest in cancer research since the demonstration that at low concentration (5–10 $\mu$M) they augment the cytotoxicity of many standard anti-cancer agents in a variety of tumour cell types (Tsuruo *et al.*, 1983*a,b*; Yalowich & Ross, 1984, 1985; Robinson *et al.*, 1985; Ince *et al.*, 1986; Merry *et al.*, 1986). Verapamil, the prototype calcium channel blocker, enhances the cytotoxic effects of both vincristine and adriamycin *in vitro* as well as *in vivo* in cells previously resistant to these drugs (Tsuruo *et al.*, 1981, 1983*a,b*). Although the precise mechanism of this increased cytotoxic effect is not completely understood, the calcium channel blockers are thought to act by blocking efflux of the chemotherapeutic agents from the cell (Tsuruo *et al.*, 1982).

At higher concentrations (10–100 $\mu$M), verapamil by itself reversibly inhibits cell growth in several human cell lines (Schmidt *et al.*, 1988). Protein synthesis, DNA synthesis and RNA synthesis are all inhibited within minutes of addition of 100 $\mu$M verapamil to the cells; removal of the drug by simple washing of the cells results in a rapid resumption of cell growth (Schmidt *et al.*, 1988). These reversible anti-proliferative effects of verapamil make it an ideal compound to study cell cycle related events.

Cell growth is controlled by a cascade of events that ultimately leads to DNA synthesis. Briefly, cell proliferation begins when growth factors interact with the cell membrane, sending a signal via inositol phospholipids to increase cytoplasmic ionised calcium by one pathway and to increase cytoplasmic pH by another pathway (Berridge, 1984). However, stimulation of protein kinase C by phorbol esters (e.g. TPA) directly causes cytoplasmic alkalinisation and in at least some cells this stimulation can by-pass the calcium pathway (Rozengurt & Mendoza, 1985). Furthermore, the involvement of c-*onc* genes in cell proliferation has been extensively documented (Kahn & Graf, 1986).

One of the first genes linked to cell growth was the proto-oncogene c-*myc*. C-*myc* gene expression is known to be linked tightly to cell proliferation, increasing 10–20 fold in cells treated with some mitogens (Kelly *et al.*, 1983). The c-*myc* gene, which is expressed in both malignant and normal cells, encodes a protein that is functionally involved in DNA synthesis (Studzinski *et al.*, 1986). This protein is believed to directly regulate the rate at which cells divide (Cole, 1986).

The purpose of this study was to determine the effects of verapamil on cell cycle transit and c-*myc* gene expression in two closely related cell lines in order to shed further light on the mechanisms of verapamil's antiproliferative effects. B10.BR normal melanocytes and B16 melanoma cells were chosen for this study because these cells exhibited markedly different responses to incubation with verapamil in preliminary experiments.

### Methods

### *Cell culture*

Murine melanoma cell lines B16 F1 and B16 F10 were obtained from ATCC. The cells were grown in RPMI medium supplemented with 10% FCS, plus penicillin, streptomycin and fungizone. Melanocytes from B10.BR mice (Tamura *et al.*, 1987) were kindly provided by Dr Ruth Halaban, Yale University, New Haven, CT. These cells were incubated in Ham's F10 medium supplemented with 15% fetal calf serum and 48 nM TPA (12-*O*-tetradecanoyl-phorbol-13-acetate), plus penicillin, streptomycin and fungizone. The growth chamber was maintained at 37°C with 5% $CO_2$. For experiments excluding TPA, cells were incubated in Ham's F10 medium without TPA for 48 h. Cells were used before their 20th generation. The potential for the cells to metastasize was tested according to Fidler & Kripke (1977). After injection of 50,000 to 100,000 cells into the tail veins of C57BL/6 mice, the B16 F10 cells form many more pulmonary metastases than the B16 F1 cells within 2–3 weeks. All experiments were performed at least twice.

### *Radioisotope incorporation*

Actively growing cells (in the exponential phase) were always used when assessing isotope incorporation, Methyl-$^3$H-thymidine was added to cell cultures at a final concentration of 0.5 $\mu$Ci ml$^{-1}$. At timed intervals, cell samples were removed after trypsinisation and added to an equal volume

Correspondence: K.R. Huber, Children's Cancer Research Laboratory, Department of Pediatrics, Division of Hematology/Oncology, School of Medicine, University of South Carolina, Columbia, SC 29208, USA.
Received 14 September 1988, and in revised form, 28 November 1988.

of cold 10% trichloroacetic acid. Precipitates were allowed to form for 30 min on ice before filtering through Whatman GF/C glass filters mounted in a vacuum manifold. After washing with cold saline solution and ethanol, the filters were dried under a heat lamp, then counted for radioactivity in Ready-Solv-MP (Beckman Instruments) with a scintillation spectrophotometer.

*Cell cycle analysis*

The nuclei isolation medium (NIM) (Thornthwaite *et al.*, 1980) contained per litre: 10 mmol phosphate buffer, 146 mmol NaCl, 1.0 mmol $CaCl_2$, 0.5 mmol $MgSO_4 \cdot 7H_2O$, 6.0 ml Nonidet NP40 (Sigma) and 700 units RNase (Sigma type 1A, boiled for 10 min to remove residual DNase activity). The DNA fluorochrome propidium iodide (PI) (Sigma) was dissolved in NIM at a concentration of 50 $\mu g\,ml^{-1}$. Monolayer cells (triplicates) were washed by rinsing with phosphate buffered saline (PBS) before the addition of NIM buffer. The nuclei were kept in NIM buffer for at least 16 h and then filtered through a 70 $\mu m$ nylon mesh. Cell cycle analyses on the PI-stained nuclei were performed on a Coulter Electronics Epics V flow cytometer (Coulter Electronics Inc., Hialeah, FL). The instrument was adjusted to achieve coefficients of variation for the nuclei in the range from 3 to 5%. The relative fluorescence intensities of 10,000 PI-stained nuclei were measured and the proportion of nuclei in G1, S and G2-M was calculated using the Para I data analysis program of the Epics flow cytometer.

*RNA and DNA isolations and hybridisations*

For RNA isolation, cells ($10^7$) were washed three times in PBS and transferred to polypropylene tubes. The cell pellet was resuspended in 0.5 ml of extraction buffer (250 mM NaCl, 50 mM Tris-hydrochloride (pH 7.4), 5 mM EDTA, 1% sodium dodecyl sulphate and 1 $mg\,ml^{-1}$ of proteinase K (Sigma)). After incubation for 30 min at 37°C the mixture was sonicated for three 5-s bursts to shear DNA, and extracted with a solution containing 0.5 ml of phenol and 0.25 ml of chloroform. The aqueous phase was extracted again with phenol-chloroform (2:1), washed twice with chloroform, and precipitated with ethanol. DNA was extracted by taking up the pellet in 2 M LiCl solution containing 10 mM EDTA. After centrifugation (10,000 r.p.m., 5 min) the RNA pellet was suspended in $H_2O$. Total RNA was denatured with 6% formaldehyde and 50% formamide, heated 5 min to 65°C, size fractionated on a 1% agarose gel containing 2.2 M formaldehyde and blotted onto nitrocellulose or nylon membranes (Hybond, Amersham) according to Thomas (1980).

For DNA preparations, cells were added to extraction buffer, then incubated overnight at 37°C. After phenol-chloroform extractions, 10 $\mu l\,ml^{-1}$ of RNase solution (100 $\mu g\,ml^{-1}$, DNase free) was added and the mixture was incubated for another 30 min. After subsequent ammonium acetate (3 M) precipitations to remove protein, the DNA was precipitated with ethanol. Aliquots were incubated with 3 units of restriction enzymes (Bam HI, Xba I, Xho I, Bgl II, Sst I) per $\mu g$ of DNA at 37°C overnight, size separated on agarose gels, and blotted on to nitrocellulose or nylon membranes according to Southern (1975).

Hybridisations were carried out using a nick translated $^{32}P$ labelled 1,000 b.p. Pst I fragment obtained from a c-*myc* cDNA clone (pM c-*myc* 54) (Stanton *et al.*, 1983). The membranes were hybridised overnight at 55°C in the presence of 10% dextran sulphate, 50% formamide, 5 × SSC, 5 × Denhardt's solution and 100 $\mu g$ calf thymus DNA $ml^{-1}$. Three post-hybridisation washes were carried out at 55°C with 0.75 M NaCl, 0.15 M Tris (pH 8), 10 mM EDTA, 25 mM $NaPO_4$ (pH 6.8), 0.1% sodiumpyrophosphate, 0.1% SDS for 1 h; 0.15 M NaCl, 30 mM Tris, 2 mM EDTA, 25 mM $NaPO_4$, 1 × Denhardt's solution, 0.1% sodiumpyrophosphate for 1 h; and 50 mM NaCl, 5 mM Tris, 0.4 mM EDTA, 0.1% sodiumpyrophosphate, 0.1% SDS for 1 h, as described by DeLeon *et al.* (1983). The washed filters were exposed to Kodak X-Omat X-ray film with intensifying screens for 18–24 h at −70°C. Filters were routinely reprobed for the efficiency of 'Northern' transfer with a probe for 18 S rRNA (Bowman *et al.*, 1981). The intensities of the autoradiographic signals were quantitated by densitometric scanning.

### Results

*C-*myc *expression in normal melanocytes and melanoma cell lines*

Experiments were first performed to establish base-line c-*myc* mRNA levels in continuously growing B10.BR melanocytes and B16 melanoma cells. Because B10.BR murine melanocytes require the addition of TPA to their medium for continuous growth (Tamura *et al.*, 1987), total RNA was extracted from cells incubated for 48 h in either the presence or absence of TPA. C-*myc* expression under these conditions was compared to that obtained in B16 cells with both a high metastatic potential (B16 F10) and low metastatic potential (B16 F1). The RNA was probed with a $^{32}P$-labelled 1,000 b.p. Pst I fragment of a c-*myc* clone. In one experiment, the filter was double probed for both c-*myc* and thymidine kinase (tk) gene expression (to confirm increased DNA synthesis). The results of this experiment, as illustrated by Figure 1 (upper panel, lanes 3, 4) show an approximate 8-fold increase in c-*myc* mRNA caused by addition of TPA. For the analysis, the filters were reprobed for 18 S rRNA as a measure of the amount of RNA per lane (the slightly lower amount of RNA in lane 4 has been corrected for by densitometric analysis). Figure 1 also demonstrates that c-*myc* mRNA expression in TPA-stimulated melanocytes (lane 4) is comparable to that obtained in both B16 F10 (lane 1) and B16 F1 melanoma cells (lane 2). Thus, actively dividing melanocytes and melanoma cells express similar amounts of c-*myc* mRNA.

**Figure 1** C-*myc* expression in B16 melanoma cells and B10.BR melanocytes. Upper panel: exponentially growing B16 F1 and F10 cells (low and high metastatic potential) were harvested, total RNA was isolated and 20 $\mu g$ each resolved on denaturing agarose gels. B10.BR cells were incubated in medium with or without TPA for 48 h before they were harvested, 20 $\mu g$ total RNA each was resolved on denaturing agarose gels. The filters were hybridised with $10^7$ c.p.m. $^{32}P$ nick translated c-*myc* cDNA insert and a thymidine kinase (TK) gene specific probe, as described in Materials and methods. Lower panel: the same filters were reprobed with $^{32}P$ labelled p5B for analysis of 18S rRNA.

**Figure 2** Restriction analysis of c-*myc* from Balb/c spleen and B16F10 melanoma cells. DNA (20 μg per lane) was digested to completion with Bam HI (lanes 1 and 6), Xba I (lanes 2 and 7), Xho I (lanes 3 and 8), Bgl II (lanes 4 and 9) and Sst I (lanes 5 and 10). Digests were fractionated on an agarose gel, transferred to Hybond membranes and hybridised with a 1,400 b.p. Xho I c-*myc* fragment as described. The size marker is a Hind III digest of phage Lambda DNA.

*Southern analysis of c-*myc *gene sequences*

We then assayed for possible rearrangements in the c-*myc* sequences in the melanoma cells that might account for the constitutive c-*myc* expression in these cells. DNA was completely digested with enzymes that have recognition sites within the c-*myc* gene and in the flanking regions as described in Methods. As can be seen in Figure 2, there are no obvious rearrangements in the c-*myc* gene in the melanoma cells compared to normal control spleen cells. Three separate experiments indicated that no amplification of the c-*myc* gene in the B16 melanoma cells had occurred.

*Effects of verapamil on cell cycle and c-*myc *expression*

To monitor the effects of verapamil on DNA distribution and c-*myc* expression, cells were incubated with verapamil for varying periods of time. Nuclei were analysed on a flow cytometer and RNA was isolated and assayed for c-*myc* mRNA. Different effects of verapamil on melanoma cells and normal melanocytes were obtained.

Incubation with 100 μM verapamil has little effect on B16 cells, the $^3$H-thymidine incorporation assay shows that DNA synthesis in B16 cells is only 10% inhibited after 3 h (Table II). Longer incubation with 100 μM verapamil transiently reduces the number of cells entering from $G_1$ into S-phase, the cells already in S-phase continue their cell cycle transit (Figure 3, Table I). Parallel to the induction of synchronised progression through the cycle there is also a concomitant increase in the expression of the c-*myc* gene (Figure 4, 12 h-lane). A high proportion of the cells in this line continues to traverse the cell cycle even after 24 h of incubation with 100 μM verapamil as evidenced by the considerable percentage of cells in S-phase (Table I, 24 h incubation with verapamil). However, drastic changes are seen once the drug is washed from the cells. Approximately 12 h after removal of verapamil the B16 cells start to produce melanin and die. Increased cell death is also reflected by the high amount of fluorescent material in front of the $G_1$ peak in the DNA histogram (Figure 3, VP release) (indicating that the cells have released nucleic acids while they deteriorated) and by about 50% lower c-*myc* mRNA levels (Figure 4). Apparently, addition of verapamil induces differentiation in these cells.

By contrast, cell growth in B10.BR normal melanocytes is blocked rapidly and reversibly by 100 μM verapamil as occurs also in other cell lines tested previously (Schmidt *et al.*, 1988). DNA synthesis is reduced by 76% within 3 h of adding 100 μM verapamil (Table II). The cell cycle phase analyses data (Figure 3 and Table I) show that the cells do not enter into S-phase in the presence of verapamil. Cells already in S-phase, however, proceed through the DNA synthesis phase. Concomitantly, the c-*myc* signal is decreased (Figure 4). After the B10.BR cells are washed free of verapamil, they rapidly resume growth as also depicted by the DNA histogram (Figure 3) and increased c-*myc* mRNA levels (Figure 4).

In summary, verapamil has different effects on the two cell lines studied. The B16 melanoma cells seem to be induced to a differentiation pathway, whereas cell growth of the B10.BR melanocytes is blocked rapidly and reversibly. C-*myc* mRNA levels parallel the distribution of cells in the cell cycle indicating that changes in c-*myc* gene expression are secondary effects of the calcium channel blocker.

**Table I** Effects of verapamil on cell cycle phase distribution in B10.BR normal melanocytes and B16 F10 melanoma cells

| *Cell line* | *Treatment* | *% of cells in $G_0$-$G_1$* | *% of cells in S* | *% of cells in $G_2$-M* |
|---|---|---|---|---|
| B16 F10 | Control | 48.3±2.8[a] | 39.5±5.6 | 12.1±2.8 |
| | 6 h VP | 44.5±1.6 | 36.4±0.9 | 19.1±1.7 |
| | 12 h VP | 65.1±1.4 | 23.5±1.2 | 11.4±0.7 |
| | 24 h VP | 66.4±3.4 | 24.8±2.3 | 8.8±2.3 |
| | Release | 56.2±3.8 | 30.4±5.8 | 13.4±2.5 |
| B10.BR | Control | 71.9±3.6 | 20.2±4.9 | 7.9±1.5 |
| | 6 h VP | 74.8±2.5 | 11.3±1.6 | 13.9±3.7 |
| | 12 h VP | 75.1±1.9 | 12.9±2.3 | 12.0±3.0 |
| | 24 h VP | 79.9±1.1 | 8.1±2.7 | 11.9±1.8 |
| | Release | 68.1±1.2 | 17.3±1.0 | 14.5±1.0 |

[a]Mean±s.d. of results obtained independently for three replicate cultures of one representative experiment. Cells were incubated 24 h with verapamil, drug was washed off, and the cells were harvested another 24 h later. Verapamil (100 μM) was added to exponentially growing cells. At the times indicated, cells were harvested and phase distributions were estimated by computer analysis of DNA histograms obtained by flow cytometry of propidium iodide-stained nuclei.

**Table II** Sensitivity of B10.BR normal melanocytes and B16 F10 melanoma cells to verapamil

| | *% inhibition of [$^3$H]-Thy incorporation* | |
|---|---|---|
| *μM verapamil* | *B10.BR* | *B16* |
| 25 | 42±7[a] | 0 |
| 50 | 59±6 | 11±7 |
| 75 | 66±2 | 12±10 |
| 100 | 76±2 | 10±9 |
| 200 | 92±3 | 38±5 |

[a]Mean±s.d. of one representative experiment ($n=5$). Exponentially growing cells were incubated for 3 h with varying concentrations of verapamil. $^3$H-thymidine (1 μCi ml$^{-1}$) was added and incorporated nucleotide measured as described in the text.

### Discussion

Our work shows that two closely related cell lines are affected differently by verapamil. The proliferation of normal B10.BR melanocytes is stopped rapidly and rever-

**Figure 3** DNA histograms of B10.BR normal melanocytes and B16 F10 melanoma cells: effect of verapamil on cell cycle transit. After various periods of incubation with 100 μM verapamil, cells were harvested and DNA histograms of 10,000 propidium iodide-stained nuclei were obtained by flow cytometry. $G_1$ peaks were translocated to channel 25 for the B10.BR cells, and to channel 50 (to indicate polyploidity) for the B16 cells. Triplicates were analysed and graphically summarised.

**Figure 4** C-*myc* expression in B10.BR normal melanocytes and B16 F10 melanoma cells: effect of verapamil on c-*myc* gene expression. Upper panels: exponentially growing cells were incubated with 100 μM verapamil for the time indicated. Cells were harvested, total RNA was isolated and 20 μg each resolved on denaturing agarose gels. The filters were hybridised with $10^7$ c.p.m. $^{32}P$ nick translated (> $10^8$ c.p.m. per μg) c-*myc* cDNA insert as described in the text. Lower panels: the same filters were reprobed with $^{32}P$-labelled p5B for analysis of 18S rRNA. The intensities of the signals are: B16 F10: control = 100%, 12 h VP = 145%, 24 h VP = 92%, VP-release = 47%; B10.BR: control = 100%, 6 h VP = 37%, 12 h VP = 44%, 24 h VP = 42%, VP-release = 58%.

sibly by 100 μM verapamil. The results with this cell line parallel our recent findings on a variety of brain tumour cell lines and normal fibroblasts (Schmidt *et al.*, 1988). In contrast to those cells, however, the B10.BR cells have been continuously incubated with the mitogen TPA. Thus, it seems that verapamil induces the same responses in cells incubated with and without this mitogen. Our results suggest that verapamil exerts its antiproliferative effect at some point beyond protein kinase C in the signal cascade that ultimately leads to DNA synthesis.

By contrast, melanoma cells continue to proliferate in the presence of 100 μM verapamil. This was the first cell line tested so far in this laboratory that continued to proliferate in the presence of verapamil without having been selected for resistance. However, addition of verapamil seems to induce a differentiation pathway because these cells start to produce melanin and die after verapamil is removed. It remains to be established whether this induction of differentiation can be exploited for *in vivo* treatment.

Since c-*myc* gene expression is known to be tightly linked to cell proliferation we were interested in the effects of verapamil on the expression of this gene. This linkage is not completely straightforward, however, because in some *in vitro* differentiation model systems c-*myc* expression is slightly increased following the induction of differentiation (Curran & Morgan, 1985; Lachman & Skoultchi, 1984), and *myc* protein levels can stay unchanged while *myc* mRNA levels are decreased (Wingrove *et al.*, 1988).

Our results show that c-*myc* mRNA levels parallel the effects of verapamil, decreasing in the melanocytes while the cells are arrested, whereas in the B16 cells which continue to proliferate c-*myc* expression is transiently increased. Subsequent Southern analyses of c-*myc* sequences did not indicate any obvious alterations in the c-*myc* gene in the melanoma cells as compared to normal spleen cells. Obviously, alterations in this gene do not seem to be responsible for the different responses of the respective cell lines to verapamil. It remains to be established whether the different levels of c-*myc* expression result from changed gene transcription or changed mRNA stability, because it is possible that verapamil affects post-transcriptional mechanisms that control the concentration of c-*myc* mRNA (Blanchard *et al.*, 1985) differently in these cells. Post-transcriptional modulation of c-*myc* mRNA can be mediated by a labile degradative protein and depends on active protein synthesis (Santos *et al.*, 1988). Inhibition of protein synthesis results in superinduction of c-*myc* mRNA. Because verapamil stops protein synthesis in cells that are arrested (Schmidt *et al.*, 1988), our data indicate that there is rather no post-transcriptional modulation in the B10.BR cells as there is no superinduction of c-*myc* mRNA. The B16 cells show a transient decline in the number of cells entering into S-phase and a concomitant synchronised progression of the cells that have been in S-phase through the cell cycle. The transient increase in c-*myc* expression in this cell line induced by verapamil appears to be a consequence of the distribution of the cells in the mitotic cycle because c-*myc* is expressed slightly higher in G1 phase of the cell cycle, particularly after induction of differentiation (Lachman *et al.*, 1985). After removal of verapamil and extensive cell death there is also a decrease in the c-*myc* signal in the B16 cells.

Thus, we conclude that the changes in c-*myc* expression in B16 and B10.BR cells induced by verapamil are secondary to other effects of the calcium channel blocker paralleling the distribution of cells in the cell cycle.

There is recent evidence that verapamil blocks $Na^+/H^+$ exchange in cultured cells thereby interfering with the alkalinisation of the cytoplasm required for proliferation to begin (Hunter *et al.*, 1986; Bhalla & Sharma, 1986). In the model of the signalling cascade that leads to DNA synthesis, alkalinisation of the cytoplasm occurs after increases in cytoplasmic calcium concentration (Berridge, 1984). As we have shown that verapamil seems to exert its effects independently of calcium fluxes (Schmidt *et al.*, 1988), protein kinase C and c-*myc* expression, interference with cytoplasmic alkalinisation might be a likely candidate for verapamil's antiproliferative effects. Work to test this possibility is currently underway in this laboratory.

This work was supported in part by ACS grant IN-107L and the South Carolina Endowment for Children's Cancer Research.

### References

BHALLA, R.C. & SHARMA, R.V. (1986). Competitive interaction of amiloride and verapamil with alpha 1-adrenoreceptors in vascular smooth muscle. *J. Cardiovasc. Pharm.*, **8**, 927.

BERRIDGE, M.J. (1984). Oncogenes, inositol lipids, and cellular proliferation. *Bio/tech*, **4**, 541.

BLANCHARD, J.M., PIECHACZYK, M., DANI, C. and 4 others (1985). C-*myc* gene is transcribed at high rate in G0-arrested fibroblasts and is post-transcriptionally regulated in response to growth factors. *Nature*, **317**, 443.

BOWMAN, L.H., RABIN, B. & SCHLESSINGER, D. (1981). Multiple ribosomal RNA cleavage pathways in mammalian cells. *Nucleic Acids Res.*, **9**, 4951.

COLE, M.D. (1986). The *myc* oncogene: its role in transformation and differentiation. *Ann. Rev. Gen.*, **20**, 361.

CURRAN, T. & MORGAN, J.I. (1985). Superinduction of c-fos by nerve growth factor in the presence of peripherally active benzodiazepines. *Science*, **229**, 1265.

DELEON, D.V., COX, K.H., ANGERER, L.M. & ANGERER, R.C. (1983). Most early-variant histone mRNA is contained in the pronucleus of sea urchin eggs. *Develop. Biol.*, **100**, 197.

FIDLER, I.J. & KRIPKE, M.L. (1977). Metastasis results from preexisting variant cells within a malignant tumour. *Science*, **197**, 893.

HUNTER, D.R., HAWORTH, R.A. GOKNUR, A.B. and 4 others (1986). Control of thallium and sodium fluxes in isolated adult rat heart cells by Anthopleurin-A, verapamil, and magnesium. *J. Mol. Cell. Cardiol.*, **18**, 1125.

INCE, P., APPLETON, D.R., FINNEY, K.J., SUNTER, J.P. & WATSON, A.J. (1986). Verapamil increases the sensitivity of primary human colorectal carcinoma tissue to vincristine. *Br. J. Cancer*, **53**, 137.

KAHN, P. & GRAF, T. (1986). *Oncogenes and growth control.* Springer-Verlag: Berlin, Heidelberg.

KELLY, K., COCHRAN, B.H., STILES, C.D. & LEDER, P. (1983). Cell-specific regulation of the c-myc gene by lymphocyte mitogens and platelet-derived growth factor. *Cell*, **35**, 603.

LACHMAN, H.M., HATTON, K.S., SKOULTCHI, A.I. & SCHILDKRAUT, C.L. (1985). C-myc mRNA levels in the cell cycle change in mouse erythroleukemia cells following inducer treatment. *Proc. Natl Acad. Sci. USA*, **82**, 5323.

LACHMAN, H.M. & SKOULTCHI, A.I. (1984). Expression of c-myc changes during differentiation of mouse erythroleukaemia cells. *Nature*, **310**, 592.

MERRY, S., FETHERSTON, C.A., KAYE, S.B., FRESHNEY, R.I. & PLUMB, J.A. (1986). Resistance of human glioma to adriamycin *in vitro*: the role of membrane transport and its circumvention with verapamil. *Br. J. Cancer*, **53**, 129.

ROBINSON, B.A., CLUTTERBECK, R.D., MILLAR, J.L. & McELWAIN, T.J. (1985). Verapamil potentiation of melphalan cytotoxicity and cellular uptake in murine fibrosarcoma and bone marrow. *Br. J. Cancer*, **52**, 813.

ROZENGURT, E. & MENDOZA, S.A. (1985). Synergistic signals in mitogenesis: role in ion fluxes, cyclic nucleotides, and protein kinase C in Swiss 3T3 cells. *J. Cell Sci.*, suppl., **3**, 229.

SANTOS, G.F., SCOTT, G.K., LEE, W.M.F., LIU, E. & BENZ, C. (1988). Estrogen-induced posttranscriptional modulation of c-myc proto-oncogene expression in human breast cancer cells. *J. Biol. Chem.*, **263**, 9565.

SCHMIDT, W.F., HUBER, K.R., ETTINGER, R.S. & NEUBERG, R.W. (1988). Antiproliferative effects of verapamil alone on human tumor cells *in vitro*. *Cancer Res.*, **48**, 3617.

SOUTHERN, E.M. (1975). Detection of specific sequences among DNA fragments separated by gel electrophoresis. *J. Mol. Biol.*, **98**, 503.

STANTON, L.W., WATTS, R. & MARCU, K.B. (1983). Translocation, breakage and truncated transcripts of c-*myc* oncogene in murine plasmacytomas. *Nature*, **303**, 401.

STUDZINSKI, G.P., BRELVI, Z.S., FELDMAN, S.C. & WATT, R.A. (1986). Participation of c-*myc* protein in DNA synthesis of human cells. *Science*, **234**, 467.

TAMURA, A., HALABAN, R., MOELLMANN, G. and 4 others (1987). Normal murine melanocytes in culture. *In Vitro Cell. Develop. Biol.*, **23**, 519.

THOMAS, P.S. (1980). Hybridization of denatured RNA and small DNA fragments transferred to nitrocellulose. *Proc. Natl Acad. Sci. USA*, **77**, 5201.

THORNTHWAITE, J.T., SUGARBAKER, E.V. & TEMPLE, W.J. (1980). Preparation of tissues for DNA flow cytometric analysis. *Cytometry*, **1**, 229.

TSURUO, T., IIDA, H., NOJIRI, M., TSUKAGOSHI, S. & SAKURI, Y. (1983*a*). Circumvention of vincristine and adriamycin resistance *in vitro* and *in vivo* by calcium influx blockers. *Cancer Res.*, **43**, 2905.

TSURUO, T., IIDA, H., TSUKAGOSHI, S. & SAKURI, Y. (1981). Overcoming of vincristine resistance in P388 leukemia *in vivo* and *in vitro* through enhanced cytotoxicity of vincristine and vinblastine by verapamil. *Cancer Res.*, **41**, 1967.

TSURUO, T., IIDA, H., TSUKAGOSHI, S. & SAKURI, Y. (1982). Increased accumulation of vincristine and adriamycin in drug-resistant P388 tumor cells following incubation with calcium antagonists and calmodulin inhibitors. *Cancer Res.*, **42**, 4730.

TSURUO, T., IIDA, H., TSUKAGOSHI, S. & SAKURI, Y. (1983*b*). Potentiation of vincristine and adriamycin effects in human hemopoietic tumor cell lines by calcium antagonists and calmodulin inhibitors. *Cancer Res.*, **43**, 2267.

WINGROVE, T.G., WATT, R., KENG, P. & MACARA, I.A. (1988). Stabilization of myc proto-oncogene proteins during friend murine erythroleukemia cell differentiation. *J. Biol. Chem.*, **263**, 8918.

YALOWICH, J.C. & ROSS, W.E. (1984). Potentiation of etoposide-induced DNA damage by calcium antagonists in L1210 cells *in vitro*. *Cancer Res.*, **44**, 3360.

YALOWICH, J.C. & ROSS, W.E. (1985). Verapamil-induced augmentation of etoposide accumulation in L1210 cells *in vitro*. *Cancer Res.*, **45**, 1651.